# The effects of organic solvents on the folding pathway and associated thermodynamics of proteins: a microscopic view

**DOI:** 10.1038/srep19500

**Published:** 2016-01-18

**Authors:** Yuqi Yu, Jinan Wang, Qiang Shao, Jiye Shi, Weiliang Zhu

**Affiliations:** 1 Drug Discovery and Design Center, CAS Key Laboratory of Receptor Research, Shanghai Institute of Materia Medica, Chinese Academy of Sciences, 555 Zuchongzhi Road, Shanghai, 201203, China; 2 UCB Biopharma SPRL, Chemin du Foriest, Braine-l’Alleud, Belgium

## Abstract

Protein folding is subject to the effects of solvation environment. A variety of organic solvents are used as additives for *in vitro* refolding of denatured proteins. Examination of the solvent effects on protein folding could be of fundamental importance to understand the molecular interactions in determining protein structure. This article investigated the folding of α-helix and β-hairpin structures in water and the solutions of two representative refolding additives (methanol (MeOH) and 1-Ethyl-3-methylimidazolium chloride (EMIM-Cl) ionic liquid) using REMD simulations. For both α-helix and β-hairpin in MeOH/water solution or α-helix in EMIM-Cl/water solution, the transient structures along the folding pathway are consistent with the counterparts in water but the relative statistical weights are changed, leading to the decrease in the overall folding free energy barrier. Accordingly, MeOH promotes the folding of both α-helix and β-hairpin but EMIM-Cl ionic liquid only promotes the folding of α-helix, consistent with experimental observations. The present study reveals for the first time the trivial effects on folding route but significant effects on folding thermodynamics from MeOH and EMIM-Cl, explaining the function of protein refolding additives and testifying the validity of the folding mechanism revealed by *in vitro* protein folding study using refolding additives.

Protein folding is a molecular self-assembly process in which a disordered polypeptide collapses to form a well-defined three-dimensional biologically functional structure. Understanding the molecular mechanism underlying protein folding is of fundamental importance to biology. A widely held view is that the evolution has selected a protein’s sequence in which the molecular interactions in native structure are mutually supportive and cooperatively lead to the functional structure, meaning that a given sequence is only consistent with a single native structure. The native structure of protein, as stated in free energy theory^1^,^2^, has the lowest energy in the vast protein conformational space. In this scenario, the energy landscape of protein folding is funnel-shaped and biased towards a single attractive basin of the native structure^1^,^2^. Any “trapping” protein conformations along the folding pathway have energetic depth smaller than the overall bias to the native structure, which guarantees that the native structure is both thermodynamically favorable and kinetically accessible.

Besides intrinsic properties of intra-protein interactions, protein folding is affected by extrinsic factors such as solvation environment as well. Any change in solvent condition might lead to the changes in several key aspects of protein stability and folding^3^,^4^. A conspicuous example is that water-soluble proteins can still fold into their native structures in specific organic solvents but the folding rates vary depending on the organic solvents used for protein solvation. Accordingly, protein folding is often investigated through *in vitro* refolding of denatured protein structure in various organic solvent solutions instead of pure water^5^,^6^. While the changes in thermodynamic and kinetic aspects of protein folding induced by organic solvents can be measured using the currently available experimental techniques, it is still a great challenge to monitor the folding process of protein in real time at the level of a single protein molecule. As a result, whether the folding route of protein is changed as a result of the change in solvation environment remains elusive, which may call into question the validity of the folding mechanism of protein revealed by *in vitro* experiments using organic solvents as refolding additives. A systematic investigation of the effects of organic solvents (working as refolding additives) on not only folding thermodynamics/kinetics but also detailed folding route becomes much necessary.

Alcohols such as methanol (MeOH) and trifluoroethanol (TFE) are typical organic solvents which enhances the stabilization of α-helical and β-hairpin secondary structures but meanwhile destabilizes the tertiary structure, generating a “molten globule” like state (a common folding intermediate state for small globular proteins)^5^,^7^. Multiple experiments and simulations^8^,^9^,^10^,^11^,^12^ further showed that MeOH and TFE promote the *in vitro* refolding of denatured α-helices and β-structures as well. Ionic liquids (ILs), molten salts usually composed of large-size organic cations (e.g., alkylsubstituted imidazolium, ammonium) and compact counterions, are another representative “organic solvent” which exert significant influence on protein folding, protein structural stability, and enzymatic activity. A great number of experiments have indicated that given suitable anions (e.g., BF_4_^−^, PF_6_^−^, and Tf_2_N^−^), ILs stabilize the native structures of proteins^13^,^14^,^15^,^16^,^17^, promote the refolding process^6^,^18^,^19^, and improve the catalytic activity of enzymes of widely diverging types^20^,^21^,^22^,^23^,^24^,^25^,^26^,^27^. Examination of the effects of abovementioned organic solvents on protein folding can serve as a useful model system for understanding the various molecular interactions in determining protein structures.

α-Helices and β-hairpins are the most common structural motifs in protein and the detailed study could shed lights on the mechanism of protein folding. A typical β-hairpin is characterized by a hydrophobic core cluster packed by hydrophobic side-chains from anti-parallel strands as well as the backbone hydrogen bond assembly along the strands. This complex balance of local and nonlocal interactions makes β-hairpins resemble the folding of globular proteins, and the relevant study provides information about early events in protein folding. Therefore, short polypeptides adopting either α-helix or β-hairpin or the combination of both structural motifs have attracted great attention in the last several decades. In the present study, using Trp-cage and the C-terminal β-hairpin (residues 41-56) from the B1 domain of protein G (GB1p) as protein models, we ran explicit solvent replica-exchange molecular dynamics (REMD) simulations to investigate atomically the folding of α-helix and β-hairpin structures in not only water but also ~45% *(v/v)* MeOH/water solution and ~3M 1-Ethyl-3-methylimidazolium chloride (EMIM-Cl) ionic liquid solution. The accuracy of multiple physics based force fields was first assessed in the simulations of proteins in water to determine the force field with best balance of the α-helix and β-hairpin tendencies. The structural characterization and energetics of the folding of Trp-cage and GB1p were then measured in all three abovementioned solutions, which provides comprehensive, atomic-level picture of the solvent effects on protein folding pathway as well as the associated folding thermodynamics.

## Results

### Force field assessment for the folding of α-helix and β-hairpin structures in water

A variety of force fields have been developed to measure the structural and dynamic properties of protein. Bias favoring either α-helical or β-hairpin secondary structure, however, has been observed among the force fields. The use of different force fields may produce different folding-related properties of protein. Multiple literatures have critically evaluated the performance of commonly used force fields in protein folding simulations, focusing on how accurately they reproduce the native structure and the dynamics of proteins, the experimental quantities relevant to protein folding^28^,^29^,^30^,^31^. In the present study, we chose several widely used AMBER force fields (namely AMBER FF94, FF96, and FF99SB-ILDN) to simulate the folding of GB1p and Trp-cage polypeptides in water solution. The most suitable force field for both α-helix and β-hairpin structures was determined through the comparison of the conformational landscapes, which was then used for the subsequent investigation of solvent effects on protein folding.

In REMD simulation, random walk in temperature space is essential for replicas to escape local minima so as to make a sufficient sampling for protein configuration space. For each simulation system under study, the representative replica is ergodic for every temperature in the desired temperature range (from 300 K to 550 K) during the simulation, showing the efficiency of REMD search (see Supplementary Figs S1 and S2 online). In addition, as shown in Supplementary Figs S3 and S4 online, the fraction of folded structure at room-temperature (300 K) keeps increasing from the very beginning of each simulation and reaches a maximum plateau eventually. Taking the simulation systems of GB1p and Trp-cage in water as examples, we calculated the time series of the fraction of all populated structures of GB1p and Trp-cage at 300 K. As shown in Supplementary Figs S5 and S6 online, all the fractions reach the plateau. All the information (Figs S1–S6) indicates the convergence of REMD simulations in the present study. It is noteworthy that under FF99SB-ILDN force field, the folded β-hairpin structure of GB1p is most populated in the equilibrium conformational ensemble in water and its population (~36% at 300 K) (Figs S3a and S5a) is very close to the experimentally measured population of ~45% by Munoz *et al*.^32^ and 30% by Fesinmeyer *et al*.^33^ On the other hand, the population of Trp-cage folded structure in water simulated by FF99SB-ILDN force field is consistent with the counterpart generated by the recent extensive conventional MD simulation of Lindorff-Larsen *et al*.^34^ using CHARMM22* force field. Both simulated population of Trp-cage are, however, relatively lower than the experimental data^35^,^36^. The discrepancy between simulations and experiments might be attributed to the small residual errors in the force field parameterization of two plentiful residues of proline and glycine in Trp-cage, which have an unusually large effect on the stability of this fold^34^.

**Figure 1:**
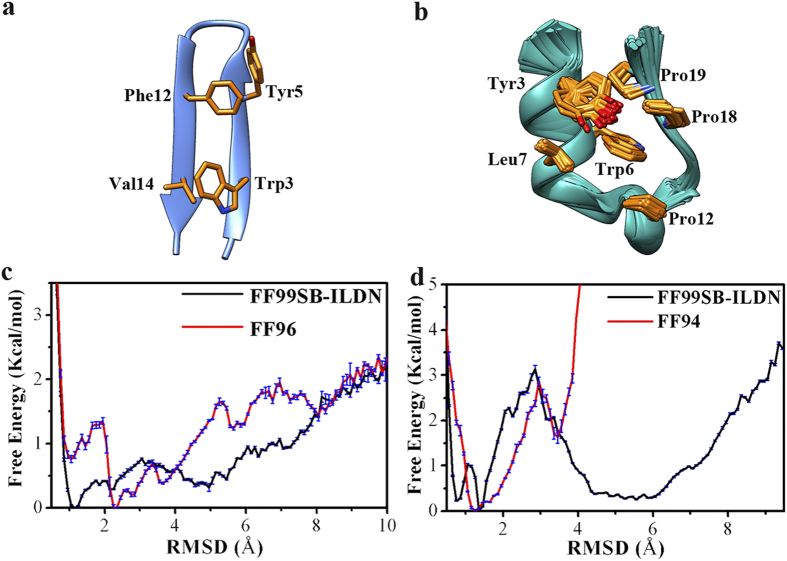
(**a,b**) NMR structures of GB1p and Trp-cage (the side-chains involved in the hydrophobic core cluster of protein are shown with yellow colored licorice representation). (**c**) One-dimensional free energy profile at 300 K as the function of the backbone RMSD with respect to the experimentally determined folded structure for GB1p in water measured by FF99SB-ILDN and FF96 force fields. (**d**) One-dimensional free energy profile at 300 K as the function of the backbone RMSD with respect to the experimentally determined folded structure for Trp-cage in water measured by FF99SB-ILDN and FF94 force fields.

Using backbone root-mean-square deviation (RMSD) with respect to the experimentally determined native structure as the reaction coordinate, the one-dimensional free energy profiles were depicted in Fig. 1 for the folding of GB1p and Trp-cage in water. The free energy landscape was calculated with the normalized probability, 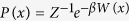 from a histogram analysis, where 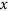 is any set of reaction coordinate(s). 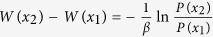 is the relative free energy or potential of mean force. It is noteworthy that all free energy profiles including Fig. 1 and the others hereinafter were calculated on the basis of the simulation data at 300 K. Accordingly, the investigation of protein folding focuses on the temperature of 300 K. For GB1p (Fig. 1c), the FF99SB-ILDN force field generates a lowest-energy basin located at the smallest RMSD (~1.0 Å) whereas the FF96 force field makes the lowest-energy basin deviate to larger RMSD (~2.2 Å). Therefore, within the similar simulation time, FF99SB-ILDN force field is better than FF96 at constructing the conformational landscape of GB1p in which the native structure is supposed to have the lowest free energy level.

**Figure 2:**
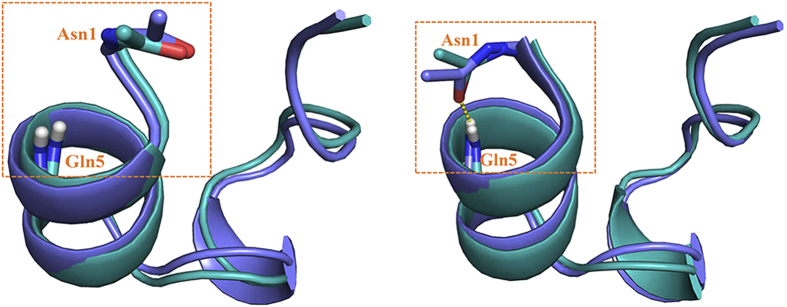
Comparison of the representative conformations (cyan colored) corresponding to the two local minima located at the low RMSD region in the free energy profile generated by FF99SB-ILDN force field (Fig. 1d) and the representative conformations of NMR structure ensembles of Trp-cage (blue colored). N-terminal backbone hydrogen bond (Asn1O-Gln5H) is highlighted with yellow square.

Meanwhile, the folding free energy profile of Trp-cage in water under FF99SB-ILDN force field (Fig. 1d) has the same shape as that generated by previous MD simulation of Lindorff-Larsen *et al*.^34^: the local minima corresponding to the folded and unfolded states are separated by a transition state (at RMSD ≈2.8 Å), implying that the folding of Trp-cage is a two-state transition (see the comparison of the folding free energy profiles in Supplementary Fig. S7 online). Ensembles of native structures of Trp-cage have been identified by previous NMR experiment, with the difference mainly at the N-terminal region: the N-terminus is flexible and the N-terminal backbone hydrogen bond (Asn1O-Gln5H) is either formed or broken^37^. Intriguingly, the low RMSD region (<2.0 Å) in the free energy profile generated by FF99SB-ILDN (Fig. 1d) is separated into two local minima. The representative conformations of the corresponding native-like structures are close to each other except that the N-terminal backbone hydrogen bond is formed in one conformation but broken in the other, perfectly reproducing the experimentally observed trivial difference among structure ensembles of Trp-cage in water (Fig. 2). On the other hand, while the free energy profile of Trp-cage generated by FF94 force field also reflects a two-state transition of protein folding, the free energy barrier from the unfolded to folded state is significantly smaller than the counterpart under FF99SB-ILDN. In addition, the free energy profile under FF94 is limited in much narrower RMSD range than the free energy profile under FF99SB-ILDN. All the information suggests that the former force field has greater bias favoring α-helix structure than the latter.

**Figure 3:**
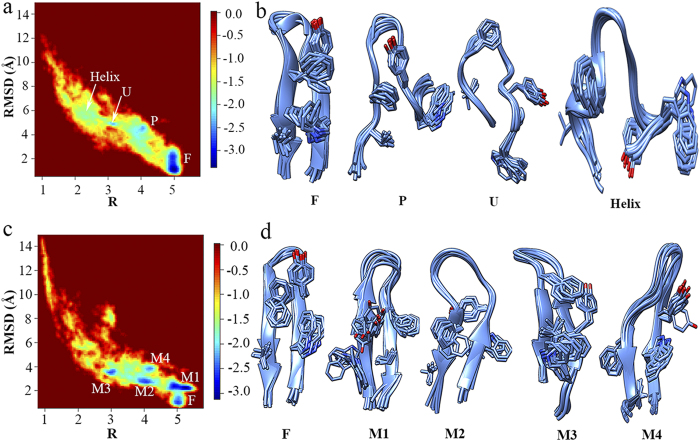
Two-dimensional free energy landscape as the function of the backbone RMSD and “R” parameter for the folding of GB1p in water and the representative conformations for all distinct states in the free energy landscape measured by (a,b) FF99SB-ILDN and (**c,d**) FF96 force fields.

To further assess the force fields in describing the folding of β-hairpin structure in water, two-dimensional free energy landscape was projected onto the backbone RMSD and a robust “R” parameter (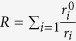, where 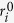 is the inter-strand C_α_-C_α_ distance in the native structure and 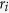 is the same distance in the simulation trajectory^38^,^39^). “R” parameter was chosen because of its ability of reflecting the formation status of the key structural elements in the folding process of β-hairpin and thus mapping the folding pathway in conformational landscape. As a comparison, radius of gyration (R_g_) was also used in combination with RMSD to draw two-dimensional free energy landscapes for GB1p in all solutions under study. The distinct states which can be clearly indicated in individual free energy landscapes of RMSD and R are undistinguished in the corresponding free energy landscapes of RMSD and R_g_ (see Supplementary Fig. S8 online). Hierarchical clustering analyses were run using *kclust* algorithm available in MMTSM Toolset^40^ to identify the representative (most populated) conformations for all distinct states indicated in Fig. 3. Four distinct states are presented in the folding free energy landscape of GB1p in water simulated by FF99SB-ILDN force field (Fig. 3a,b): the folded (F) state containing all native structural elements, the partially folded (P) state in which only the two turn-neighboring hydrophobic side-chains (Tyr5 and Phe12) are packed and the surrounding backbone hydrogen bonds are formed, the unfolded (U) state with relatively extended structure, and a state adopting mainly α-helical structure which is thus defined as Helix state here. The connection of these distinct states maps the folding pathway of GB1p. The free energy landscape of GB1p in water simulated by FF96 force field (Fig. 3c,d) is, however, much more complex: besides the folded (F) state, multiple misfolded β-hairpin structures (M1-M4) also exist (see the representative conformations in Fig. 3d and the corresponding backbone hydrogen bond assembly for all states in Supplementary Table S3 online). The formation of multiple β-hairpin structures could be attributed to the strong β-structure bias of FF96 force field.

**Figure 4:**
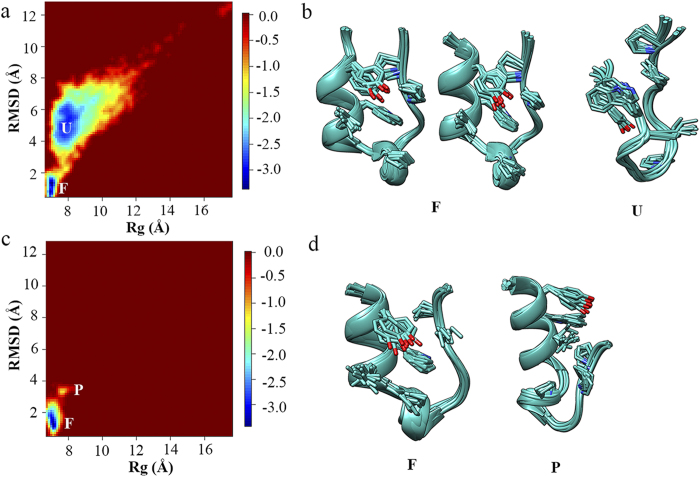
Two-dimensional free energy landscape as the function of the backbone RMSD and R_g_ for the folding of Trp-cage in water and the representative conformations for all distinct states in the free energy landscape measured by (a,b) FF99SB-ILDN and (**c,d**) FF94 force fields.

The two-dimensional free energy landscape was projected onto the backbone RMSD and R_g_ for Trp-cage in water to show the force field effects on the folding of α-helix (“R” parameter is not suitable for α-helix structure). As shown in Fig. 4, two distinct states corresponding to the folded (F) and unfolded (U) states are presented in the free energy landscape simulated by FF99SB-ILDN force field. The representative conformations of the former state are consistent with the NMR experimentally measured native structure ensembles (Fig. 2). In contrast, no secondary structure element is formed in the representative conformation of the latter state (Fig. 4b). On the other hand, the α-helix structure is well formed in both distinct states in the free energy landscape simulated by FF94 force field, making one state as the folded state and the other more likely as the partially folded (P) state, which, again, indicates strong α-helix bias of FF94 force field. In summary, among all force fields under study, FF99SB-ILDN is the most suitable force field for mimicking the folding of both α-helix and β-hairpin structures.

### Methanol promoting the folding of both α-helix and β-hairpin Structures

Next, the folding of GB1p and Trp-cage in ~45% (*v/v*) MeOH/water and 3M EMIM-Cl/water solutions was simulated using FF99SB-ILDN force field and REMD methodology, respectively. The one-dimensional free energy profile (see Supplementary Fig. S9 online) indicates that the folded state of GB1p in MeOH/water solution has the lowest energy level along the reaction coordinate. The transient states in the folding pathway of GB1p in MeOH/water have higher free energy level (relative to the folded state) than the counterparts of GB1p in water. As a result, the folding free energy barrier of GB1p (*ΔG*_f_*) is ~0.27 kcal/mol in MeOH/water solution, smaller than the value of 0.40 kcal/mol in water, suggesting that methanol promotes the folding of β-hairpin structure by decreasing the folding free energy barrier. This result is consistent with the observations in multiple experiments. For instance, using circular dichroism (CD) and NMR spectra, Searle *et al*. found that the addition of methanol or TFE promotes the native-like structure formation of the N-terminal hairpin of ferredoxin I which adopts dominantly random coil structure in pure water^9^. In addition, the NMR experiment by Platt *et al*.^41^ observed that the folding of the β-hairpin structure of a mutant of yeast ubiquitin is accelerated in low-concentrated methanol and particularly TFE aqueous solutions.

**Figure 5:**
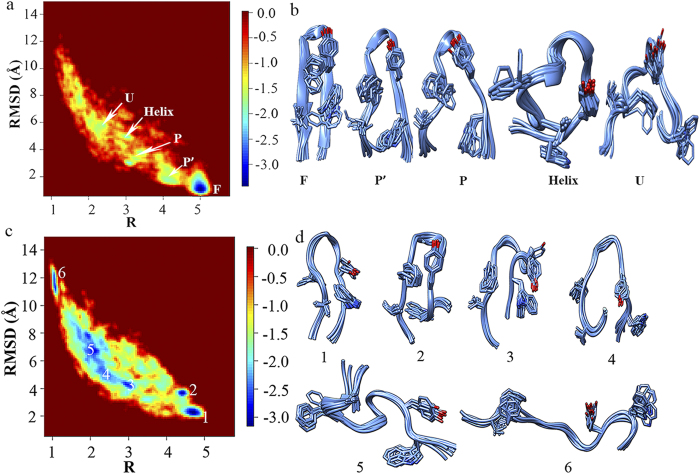
(**a,b**) Two-dimensional free energy landscape as the function of the backbone RMSD and “R” parameter for the folding of GB1p in MeOH/water solution measured by FF99SB-ILDN force field and the representative conformations for all distinct states in the free energy landscape. (**c,d**) Two-dimensional free energy landscape as the function of the backbone RMSD and “R” parameter for the folding of GB1p in EMIM-Cl/water solution measured by FF99SB-ILDN force field and the representative conformations for all distinct states in the free energy landscape.

**Figure 6:**
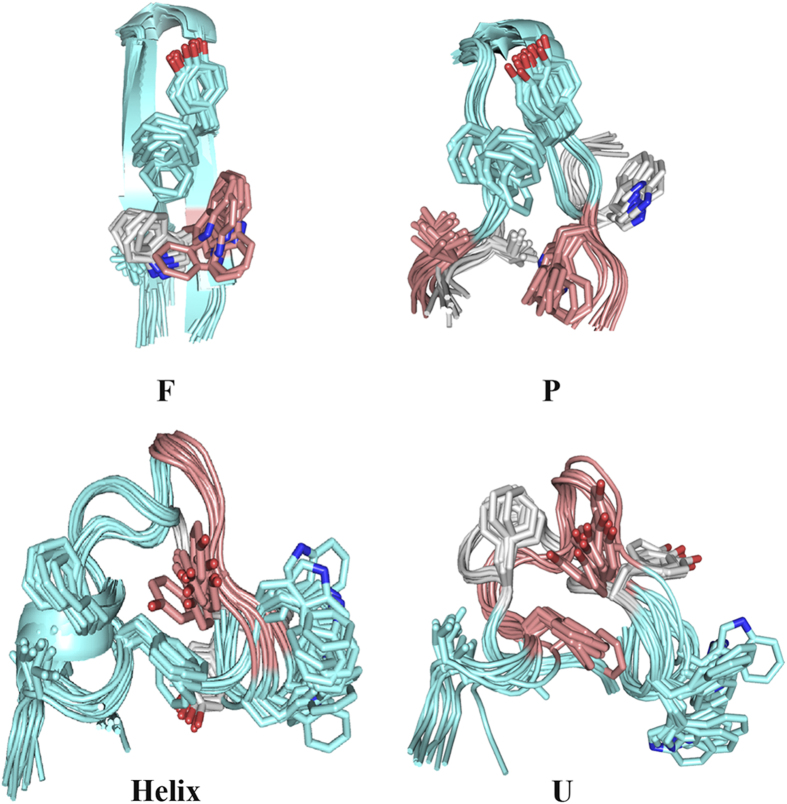
Comparison of the representative conformations of all distinct states of GB1p in MeOH/water solution and water. For each state, the regions of GB1p sharing similar conformations in two solutions are shown in green color whereas the regions having different structural features are shown in pink and grey colors in MeOH/water and water, respectively.

The four distinct states (F, P, U, and Helix states) which are presented in the two-dimensional folding free energy landscape of GB1p in water (Fig. 3a,b) can be also seen in the free energy landscape of GB1p in MeOH/water solution (Fig. 5a,b). The representative conformations of abovementioned states in MeOH/water solution are superposed onto the counterparts in water, respectively (Fig. 6). One can see that the structure of each state is largely consistent in the two solutions (e.g., the F state has only trivial difference in the orientation of Trp3 side-chain, the P state has the structure difference only in the flapping terminal regions, the U and Helix states have the structure difference mainly in the central region). In addition, another partially folded (P’) state is also presented, which has structural feature even closer to the native state than the P state. The presence of the P’ state might be attributed to methanol induced stabilization of native-like structure of β-hairpin.

**Figure 7:**
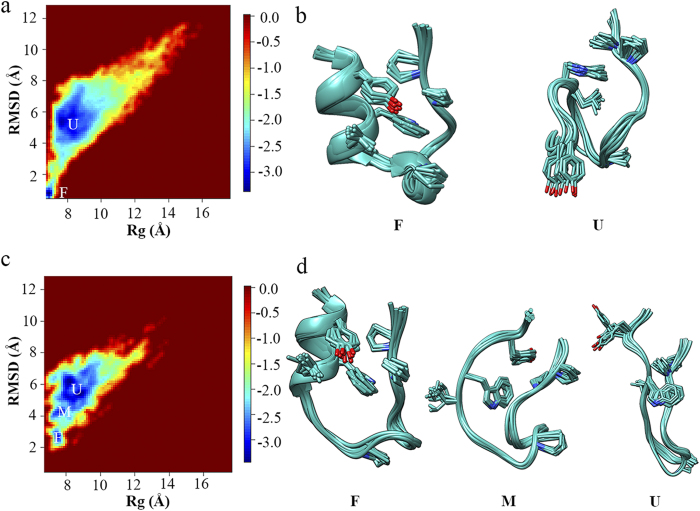
(**a,b**) Two-dimensional free energy landscape as the function of the backbone RMSD and R_g_ for the folding of Trp-cage in MeOH/water solution measured by FF99SB-ILDN force field and the representative conformations for all distinct states in the free energy landscape. (**c,d**) Two-dimensional free energy landscape as the function of the backbone RMSD and R_g_ for the folding of Trp-cage in EMIM-Cl/water solution measured by FF99SB-ILDN force field and the representative conformations for all distinct states in the free energy landscape.

On the other hand, the two-dimensional free energy landscape for Trp-cage in MeOH/water solution (Fig. 7a) has similar feature as that for Trp-cage in water except that the folding free energy barrier between the folded and unfolded states is smaller (*ΔG*_f_* is 2.44 kcal/mol in MeOH/water solution and 2.66 kcal/mol in water), suggesting that methanol could promote the folding of α-helix structure as well. The representative conformation of the unfolded state in MeOH/water is similar to that in water except that the N-terminal region is pointed to opposite direction relative to the C-terminus (see Supplementary Fig. S10 online). Interestingly, the representative conformation of the folded state of Trp-cage in MeOH/water has the N-terminal backbone hydrogen bond well formed, indicating that methanol enhances the stability of native structure of Trp-cage.

### EMIM-Cl ionic liquid promoting the folding of α-helix but not β-hairpin

Multiple ionic liquids have been reported to effectively promote the *in vitro* refolding of denatured proteins. Buchfink *et al*. tested a series of EMIM^+^ salts as protein refolding enhancers and observed that the recombinant plasminogen activator rPA has the highest refolding yield in 3 M EMIM-Cl ionic liquid solution^6^. The CD spectroscopy experiment by Huang *et al*. observed that the α-helix structured short peptides AKA_2_ and Trp-cage can fold into their native structures in neat ionic liquid of 1-butyl-1-methylpyrrolidinium bis(trifluoromethylsulfony)imide (C_4_mpy-Tf_2_N) and the formed native structures persist to very high temperature (up to 96 °C) whereas the β-hairpin structured peptide TRPZIP4 is largely destabilized in the same ionic liquid medium^42^.

The two-dimensional free energy landscape of Fig. 5c indicates that various structures from fully extended to compact ones consist of the equilibrium conformational ensemble of GB1p in EMIM-Cl/water solution. Nevertheless, even the compact structure which has the lowest RMSD in the free energy landscape (state 1 in Fig. 5c,d) is deviated from the native β-hairpin structure of GB1p: although the native β-turn configuration is formed, neither the cross-strand hydrophobic core cluster is well packed nor the backbone hydrogen bond assembly along the anti-parallel strands is formed in state 1. As a result, GB1p cannot fold spontaneously into its exact native structure in EMIM-Cl ionic liquid solution.

**Figure 8:**
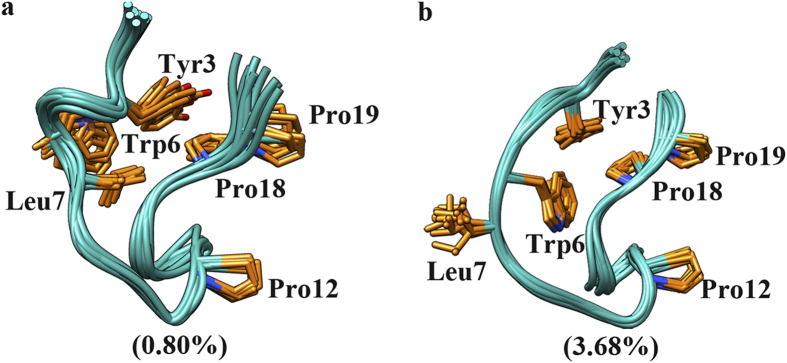
Representative conformations of the misfolded state observed in the simulations of Trp-cage in (a) water and (**b**) EMIM-Cl ionic liquid solution, along with the population in the equilibrium conformational ensembles in the corresponding solutions.

Unlike GB1p, Trp-cage can fold into its native-like structure in the same ionic liquid solution. In comparison to the native-like structures adopted in water and MeOH/water, the native-like structure adopted in EMIM-Cl/water solution is more deviated from the NMR experimentally measured native structure. Nevertheless, the key native structural elements of Trp-cage are still formed: the N-terminal helix is well folded, the C-terminal polyproline II region (including Pro18 and Pro19) is properly packed against Leu2, Tyr3, and Trp6 at the N-terminus (see the representative conformation of F state in Fig. 7d). The main deviation of the native-like structure in EMIM-Cl/water solution is that Pro12 within the 3_10_-helix (residues of Gly11-Ser14) is failed to pack with Trp6. In addition, the unfolded state of Trp-cage in EMIM-Cl/water solution has similar structure feature as the counterparts in water and MeOH/water solution. An additional state is presented between the folded and unfolded states, which contains no secondary structure content and is composed by mainly non-native intra-protein interactions as well as a small fraction of native hydrophobic contacts (Tyr3-Pro19 and Trp6-Pro18) (Fig. 8). As a result, this state is treated as a misfolded state here. The similar misfolded structure can be also seen in the equilibrium conformational ensembles in water and MeOH/water solution but with extremely low population. The overall folding free energy landscape of Trp-cage in the EMIM-Cl/water solution is flatter than that of Trp-cage in water and the folding free energy barrier from unfolded to folded state in the former case is lower than the latter (ΔG_f_* is 1.65 kcal/mol in EMIM-Cl/water solution and 2.66 kcal/mol in water).

**Figure 9:**
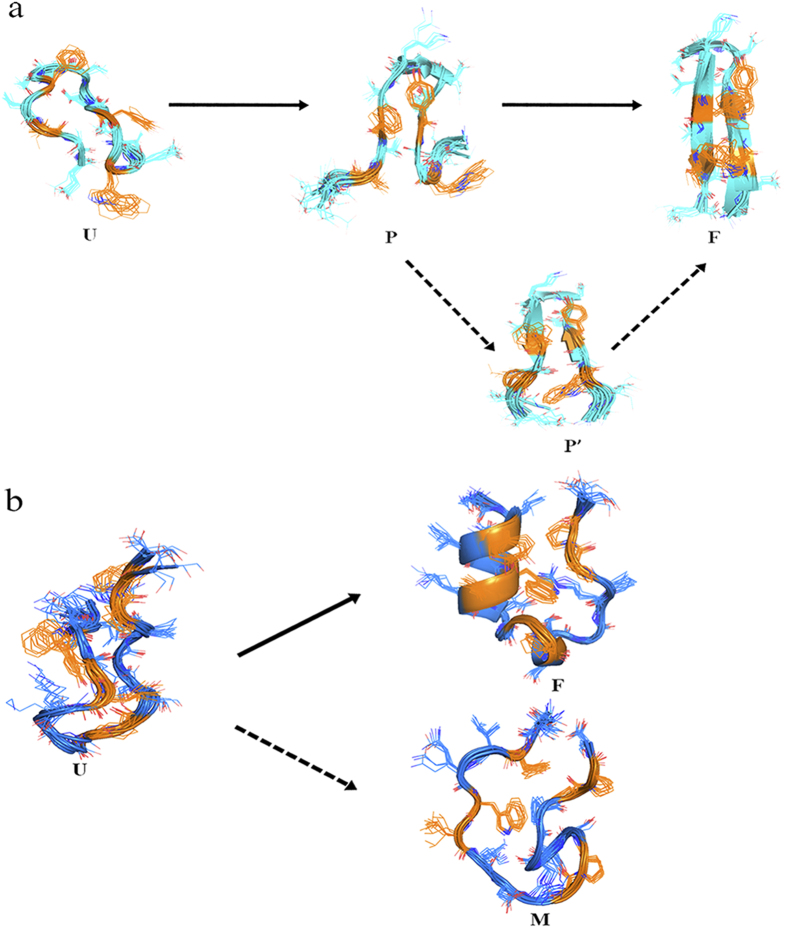
Schematics of the folding pathway of (**a**) GB1p and (**b**) Trp-cage. For clarity, residues involved in the hydrophobic core cluster of protein are colored in yellow.

Combining Figs 3, 4, 5, 6, 7, 8, one can see that methanol and EMIM-Cl ionic liquid have different influence on the folding of α-helix and β-hairpin structures: while methanol promotes the folding of both α-helix and β-hairpin structures, EMIM-Cl ionic liquid can only promote the folding of α-helix but hinder the folding of β-hairpin indeed. In the case of the protein folding promoted by either solvent, the folding free energy barrier is decreased and meanwhile the detailed folding pathway is not significantly influenced. As shown in Fig. 9a, along the folding pathway of GB1p, the unfolded structure folds into a partially folded structure (or multiple partially folded structures in MeOH/water solution) in which the cross-strand hydrophobic core cluster is partially packed and the neighboring backbone hydrogen bonds are formed. The further folding of GB1p to the native β-hairpin structure includes the well packing of cross-strand hydrophobic core cluster and the complete formation of all backbone hydrogen bonds. The folding of β-hairpin thus consists of the sequential formation of native structural elements. In contrast, for α-helical structure with a lower degree of cooperativity such as Trp-cage, the folding is a typical two-state transition. All native structural elements are formed in single transition from the unfolded to folded states. An insignificant “misfolding” seems to hide behind experimentally detectable folding of Trp-cage and the presence of EMIM-Cl ionic liquid could slightly increase the probability of such misfolding.

## Discussion

Protein folding is a consequence of complex inter-residue interactions, which should be mutually supportive and cooperatively lead to thermodynamically stable native structure^2^. A variety of experiments have indicated that the composition of the equilibrium conformational ensemble of protein folding is complex: not only the unfolded and folded states but also transiently populated “trapping” states (e.g., intermediate state) could exist^43^,^44^,^45^,^46^. By rapidly forming compact intermediate states, proteins could be driven along a preferred folding route toward their native structures and thus avoid time consuming long-range conformational search^47^. Discriminating the structural and energetic properties of various “trapping” conformations in the folding conformational ensemble sheds light on the molecular mechanism of protein folding.

Through a series of REMD simulations, the conformational landscapes of two protein models (namely GB1p and Trp-cage) were explored for their folding in water, ~45% MeOH/water, and ~3M EMIM-Cl/water solutions in the present study. The concentration of hydrated EMIM-Cl ionic liquid solution was set according to the experimental observation that EMIM-Cl is most efficient in promoting the refolding of denatured protein at 3M concentration^6^. The concentration of methanol in aqueous solution was chosen from the concentration range which has been often used in the experimental studies of methanol-induced protein conformational transition *in vitro*^48^,^49^. The relative statistical weights of various structures in the folding free energy landscape were properly estimated, providing comprehensive information of the folding mechanism of proteins in three solutions under study.

It is observed that methanol promotes the folding of both α-helix and β-hairpin structures and meanwhile EMIM-Cl ionic liquid can only promote the folding of α-helix but not β-hairpin, which are in great agreement with the experimental observation for the two organic solvents^6^,^8^,^9^,^10^,^11^,^12^,^18^,^19^. The detailed analysis reveals that for both α-helix and β-hairpin structures in MeOH/water solution or α-helix in EMIM-Cl/water solution, the transient structures along the folding pathway is maintained as in pure water whereas the relative statistical weights are changed. As a result, the folding route is maintained but the overall folding free energy barrier is decreased and accordingly the folding of specific proteins is promoted in MeOH/water and EMIM-Cl/water solutions. Various organic solvents are used as refolding additives in *in vitro* protein folding experiments but whether the organic solvents change the folding route has been not investigated deeply. The present microscopic view of the protein folding in the presence of representative protein refolding additives (methanol and EMIM-Cl ionic liquid) reveals that organic solvents can have trivial effects on folding route but significant effects on folding thermodynamics, supporting the use of refolding additives in protein folding investigation.

## Methods

All-atom molecular dynamics simulations were performed using AMBER11 package. AMBER FF99SB-ILDN^50^, FF96^51^, and FF94^52^ force fields were used to model protein atoms, TIP3P explicit solvent model^53^ was used to mimic water molecules explicitly. In addition, the parameters of the force fields concerning methanol were taken from Caldwell and Kollman^54^ and the parameters for EMIM^+^ cation were taken from Canongia Lopes *et al*.^55^ For each simulation system, a relatively extended structure of either Trp-cage or GB1p without any native structural element was placed into a cubic box containing a plenty of water (and MeOH or EMIM-Cl) molecules. Suitable counterions were then added into each system to balance the charge of protein. The detailed simulation parameters including the number of solvent molecules, box size, and simulation time for all simulation systems were organized in Supplementary Table S1 online.

For each system, REMD simulation^56^ was run in the NVT ensemble. 38 ~ 40 replicas were used at temperatures ranging from 300 K to 550 K. The number of replicas were determined by the webserver (http://folding.bmc.uu.se/remd/)^57^, and the temperature distribution along the replicas was optimized using the algorithm of Nadker and Hansmann^58^ to give a theoretical acceptance probability of 20%. The detailed temperature distribution for each system was listed in Supplementary Table S2 online. Exchanges between neighboring replicas were attempted every 1000 steps. The SHAKE algorithm^59^ was applied to constrain the bonds connecting hydrogen atoms, allowing us to use 2.0 fs as the time step. Particle mesh Ewald (PME) was applied to handle the long-range electrostatic interactions and the non-bonded cutoff of 10 Å was used^60^. The Langevin dynamics with a collision frequency of 3.0 ps^−1^ was adopted to control the temperature of the system. The coordinates were saved every 1000 steps. The simulation time per simulation system is accumulated to 2.0 ~ 6.0 μs.

## Additional Information

**How to cite this article**: Yu, Y. *et al*. The effects of organic solvents on the folding pathway and associated thermodynamics of proteins: a microscopic view. *Sci. Rep.* **6**, 19500; doi: 10.1038/srep19500 (2016).

## Supplementary Material

Supplementary Information
