# Supplementary material for: The effects of organic solvents on the folding pathway and associated thermodynamics of proteins: a microscopic view
Source: Sci Rep. 2016 Jan 18;6:19500. doi: 10.1038/srep19500 (PMC4726029; doi:10.1038/srep19500)
Supplement: Supplementary Information [file srep19500-s1.pdf]

Supplementary information

The effects of organic solvents on the folding pathway and associated thermodynamics of proteins: a microscopic view

Yuqi Yu[1], Jinan Wang[1], Qiang Shao[1*], Jiye Shi[2*], Weiliang Zhu[1*]

Drug Discovery and Design Center, Shanghai Institute of Materia Medica, Chinese Academy of Sciences, 555 Zuchongzhi Road, Shanghai, 201203, China

[1]Drug Discovery and Design Center, CAS Key Laboratory of Receptor Research, Shanghai Institute of Materia Medica, Chinese Academy of Sciences, 555 Zuchongzhi Road, Shanghai, 201203, China

[2]UCB Biopharma SPRL, Chemin du Foriest, Braine-l'Alleud, Belgium

*To whom correspondence should be addressed. Qiang Shao, Tel: +86 21 50806600-1304, E-mail: qshao@mail.shcnc.ac.cn; Jiye Shi, Jiye.Shi@ucb.com; Weiliang Zhu, wlzhu@mail.shcnc.ac.cn

Table S1. Simulation parameters.

Simulation System	Force Field	N_{water}	N_{MeOH} ($N_{EMIM\text{-}Cl}$)	Replicas of REMD	Time per Replica (ns)	Box size (Å^3)
GB1p in water	FF99SBildn	1979	-	38	80	44.2×36.4×38.6
	FF96	1979	-	38	80	44.2×36.4×38.6
GB1p in MeOH/water	FF99SBildn	1200	250	38	52	37.7×38.4×38.6
GB1p in EMIM-Cl/water	FF99SBildn	1269	100	38	116	40.7×37.4×41.5
Trp-cage in water	FF99SBildn	2061	-	40	116	40.1×40.5×40.3
	FF94	2687	-	40	100	42.8×45.6×39.4
Trp-cage in MeOH/water	FF99SBildn	1199	250	38	150	37.5×38.2×38.4
Trp-cage in EMIM-Cl/water	FF99SBildn	1193	100	38	98	39.5×40.0×39.0

Table S2. Temperature distribution in REMD simulation systems under study.

Simulation System	Temperature Range (K)
GB1p in water<br>GB1p in MeOH/water<br>GB1p in EMIM-Cl/water<br>Trp-cage in MeOH/water<br>Trp-cage in EMIM-Cl/water	300.10 304.95 309.99 315.05 320.32 325.61<br>331.00 336.00 342.00 347.45 353.65 359.30<br>365.30 371.33 377.00 383.80 389.90 396.34<br>402.50 409.00 416.31 423.18 430.17 437.25<br>444.50 451.90 459.20 467.30 474.30 482.48<br>489.80 498.51 506.70 515.10 523.62 532.25<br>541.00 550.00
Trp-cage in water<br>(FF99SB-ILDN)	300.00 304.00 309.00 314.00 319.15 324.20<br>329.32 334.48 339.72 345.04 350.44 355.93<br>361.51 367.17 372.92 378.76 384.70 390.72<br>396.84 403.06 409.37 415.70 422.30 428.98<br>435.50 442.20 449.18 456.25 463.30 470.70<br>477.95 485.20 493.00 500.25 508.08 516.10<br>524.30 532.70 541.25 550.00
Trp-cage in water (FF94)	280.00 284.10 288.20 292.40 296.70 301.10<br>305.60 310.20 314.90 319.70 324.60 329.60<br>334.70 340.00 345.40 351.00 356.60 362.50<br>368.40 374.60 380.90 387.30 394.00 400.80<br>407.80 415.10 422.50 430.10 438.00 446.00<br>454.30 462.80 471.60 480.60 489.80 499.30<br>509.00 519.00 529.20 539.70

Table S3. Intra-protein backbone hydrogen bonds in the folded and misfolded states of GB1p in water simulated by FF96 force field.

HBs	F	M1	M2	M3	M4
HB1	Thr15O-Glu2H	Glu16O-Gly1H	Thr15O-Gly1H	Thr15O-Thr4H	Thr15O-Gly1H
HB2	Glu2O-Thr15H	Gly1O-Glu16H	Gly1O-Thr15H	Thr4O-Thr15H	Thr15O-Glu2H
HB3	Thr13O-Thr4H	Val14O-Trp3H	Thr13O-Trp3H	Thr13O-Asp6H	Glu2O-Thr15H
HB4	Thr4O-Thr13H	Trp3O- Val14H	Trp3O-Thr13H	Thr13O-Asp7H	Glu2O-Val14H
HB5	Thr11O-Asp6H	Phe12O-Tyr5H	Thr11O-Tyr5H	Asp7O-Thr13H	Phe12O-Thr4H
HB6	Asp6O- Thr11H	Tyr5O-Phe12H	-	-	Thr4O-Thr11H
HB7	-	-	-	-	Thr9O-Asp6H

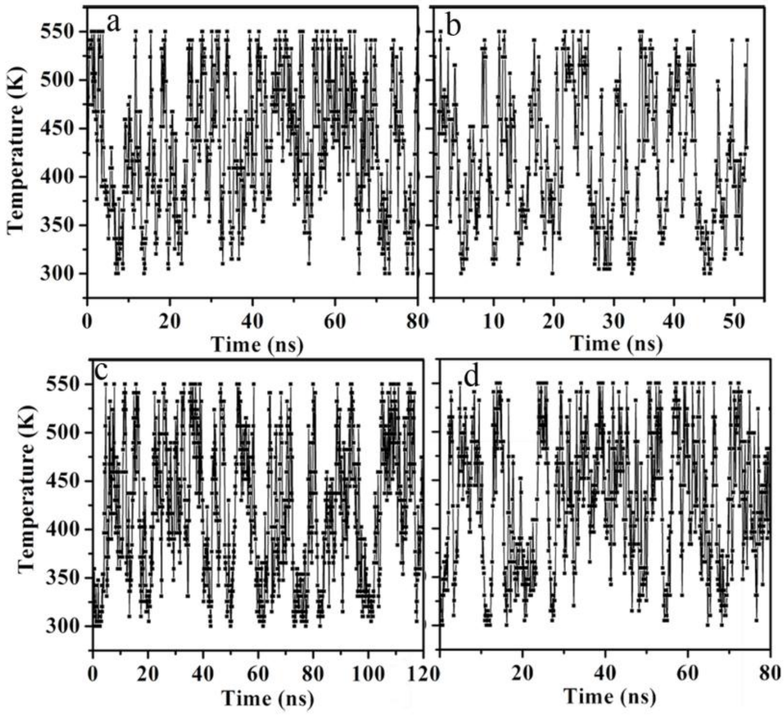

Figure S1. Temperature histories of representative replicas in the REMD simulations of (a-c) GB1p in water, MeOH/water, and EMIM-Cl/water solutions under FF99SB-ILDN force field, and (d) GB1p in water under FF96 force field. For clarity, we sampled the data every 100 exchanges.

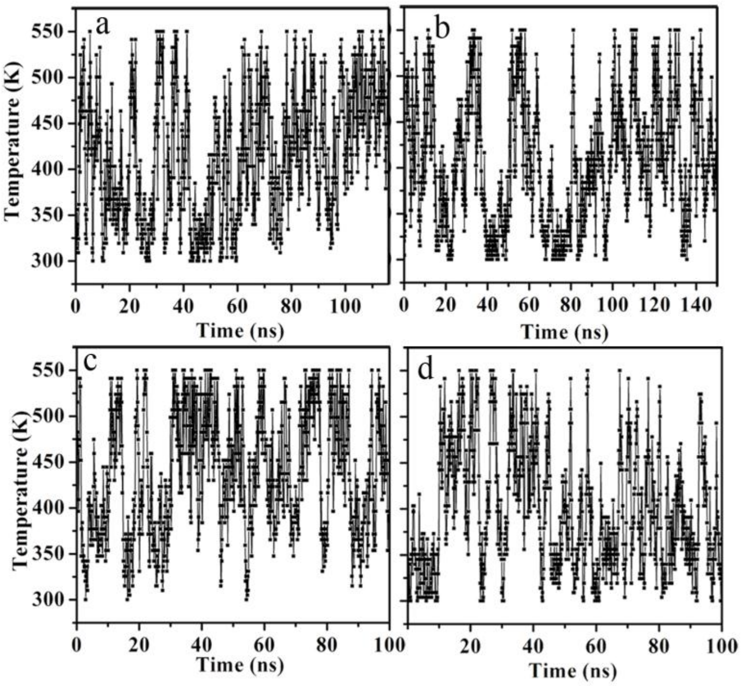

Figure S2. Temperature histories of representative replicas in the REMD simulations of (a-c) Trp-cage in water, MeOH/water, and EMIM-Cl/water solutions under FF99SB-ILDN force field, and (d) Trp-cage in water under FF94 force field. For clarity, we sampled the data every 100 exchanges.

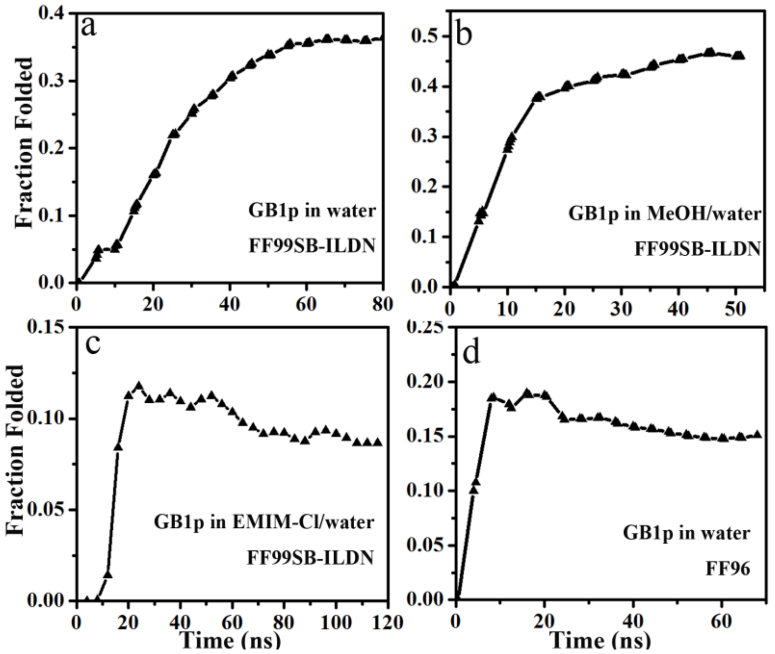

Figure S3. Time series of the fraction of folded state of GB1p at 300 K in (a) water under FF99SB-ILDN force field, (b) MeOH/water under FF99SB-ILDN force field, (c) EMIM-Cl/water under FF99SB-ILDN force field, and (d) water under FF96 force field, respectively.

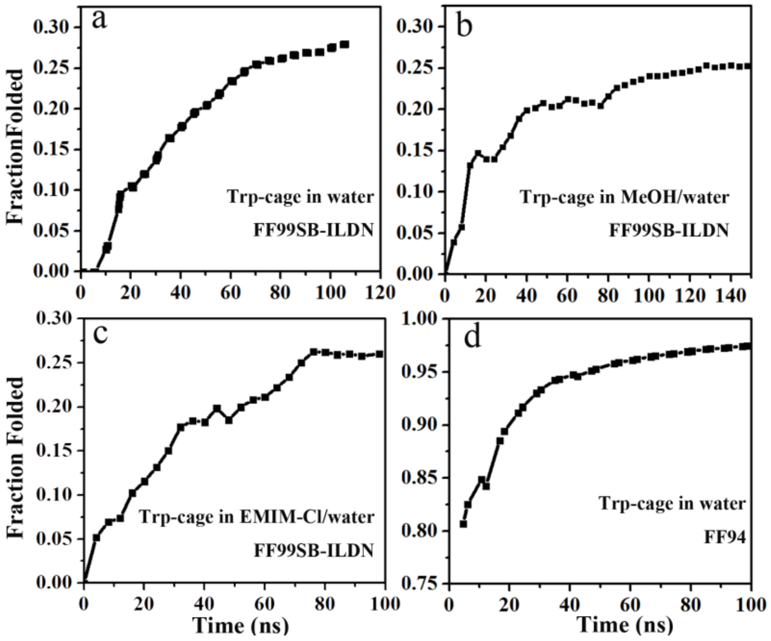

Figure S4. Time series of the fraction of folded state of Trp-cage at 300 K in (a) water under FF99SB-ILDN force field, (b) MeOH/water under FF99SB-ILDN force field, (c) EMIM-Cl/water under FF99SB-ILDN force field, and (d) water under FF94 force field, respectively.

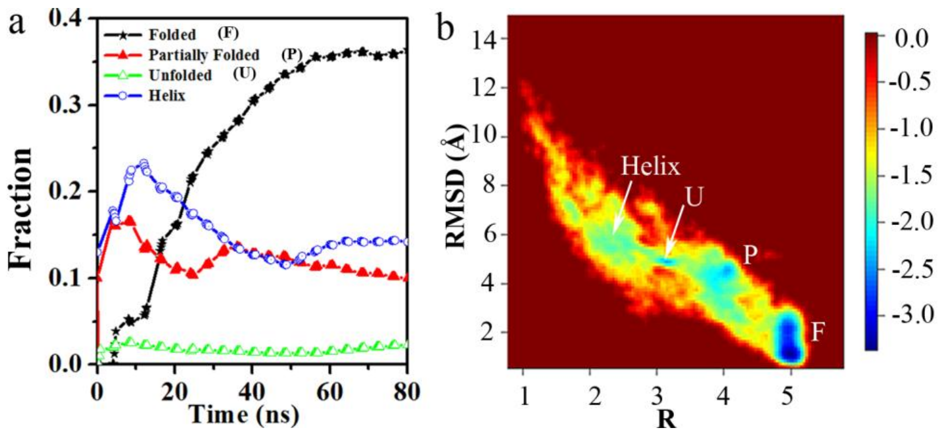

Figure S5. (a) Time series of the fraction of all distinct states in the folding pathway of GB1p in water and at 300 K as simulated by FF99SB-ILDN force field. (b) Coordinate diagram for all states (folded, partially folded, unfolded, helix states) of GB1p in two-dimensional free energy landscape at 300 K.

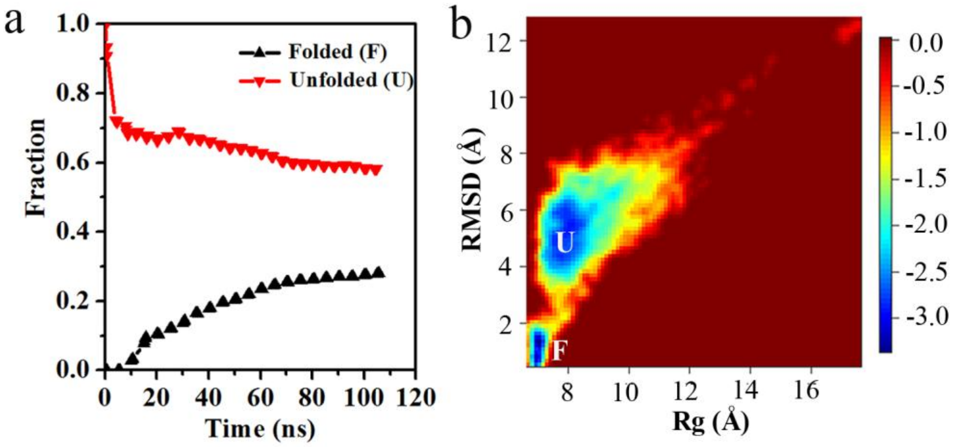

Figure S6. (a) Time series of the fraction of all distinct states in the folding pathway of Trp-cage in water and at 300 K as simulated by FF99SB-ILDN force field. (b) Coordinate diagram for all states (folded and unfolded states) of Trp-cage in two-dimensional free energy landscape at 300 K.

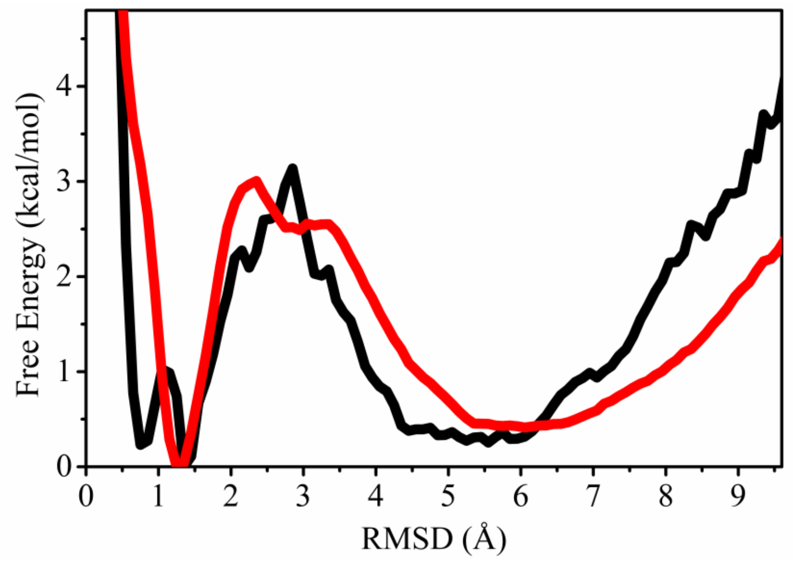

Figure S7. Comparison of one-dimensional free energy profiles as the function of backbone RMSD for GB1p in water simulated in the present REMD simulation using FF99SB-ILDN force field (black line) and in the conventional MD simulation using CHARMM22* force field by Lindorff-Larsen *et al.*[34] (red line).

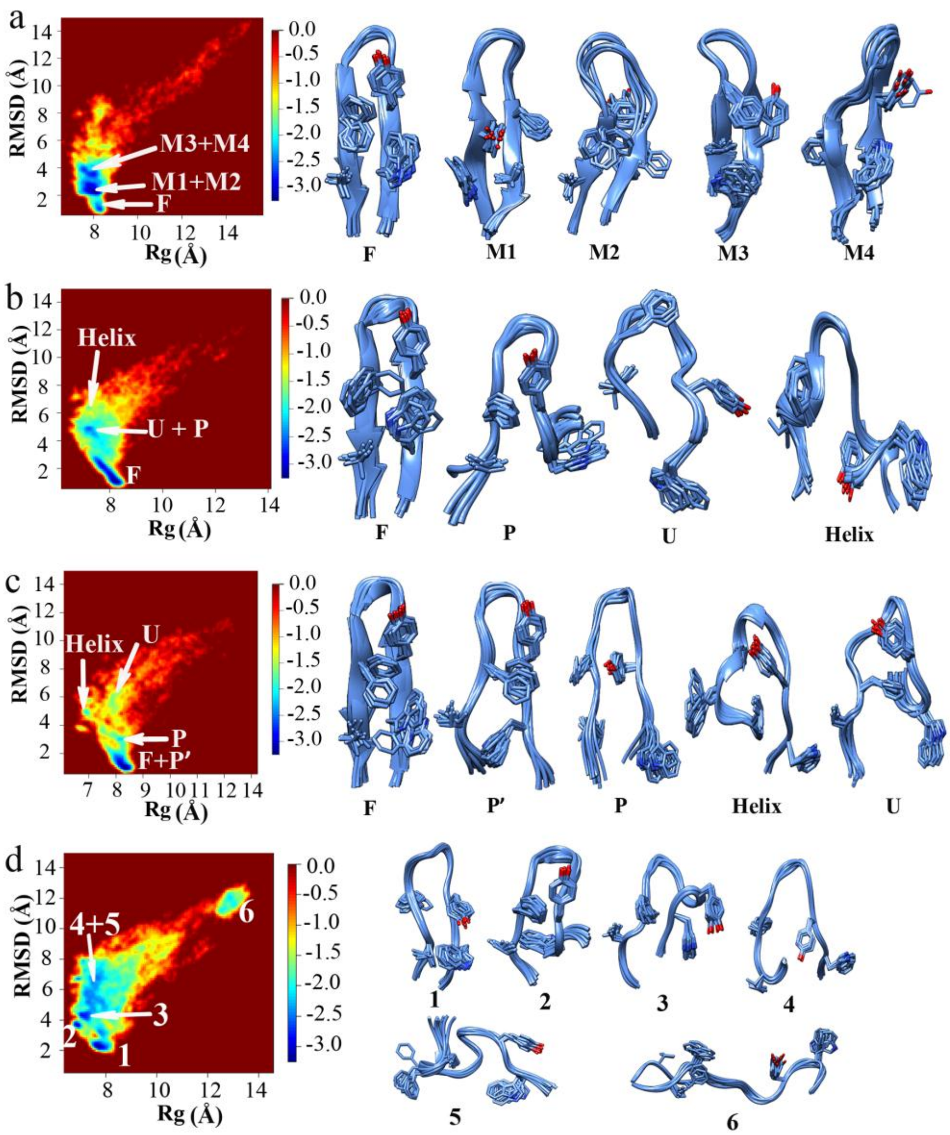

Figure S8. Two-dimensional free energy landscapes as the function of the backbone RMSD and Rg for the folding of GB1p in (a) water under FF96 force field, (b-d) water, MeOH/water, and EMIM-Cl/water under FF99SB-ILDN force field. The representative conformations for all distinct states are shown at right panel.

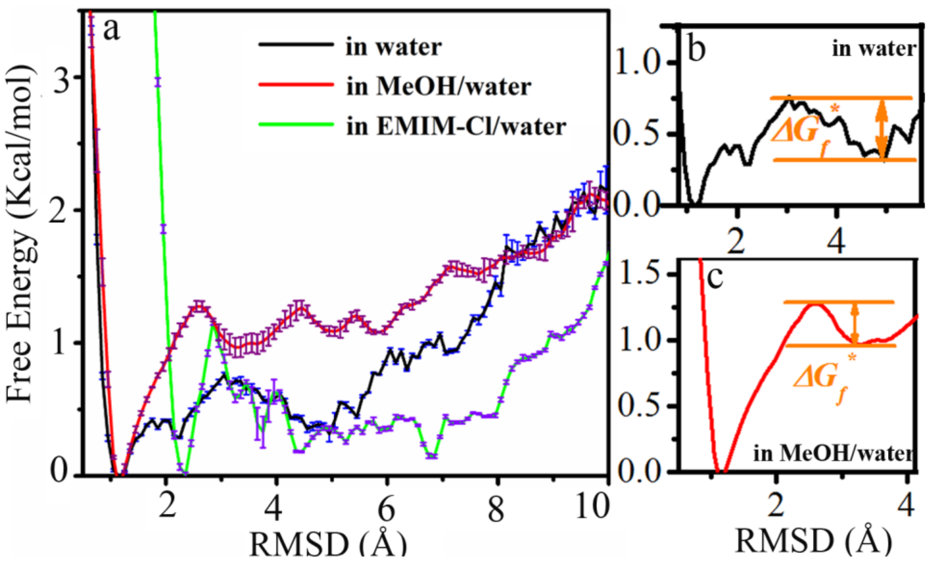

Figure S9. (a) One-dimensional free energy profiles as the function of the backbone RMSD for GB1p in water, MeOH/water, and EMIM-Cl/water solutions as simulated by FF99SB-ILDB force field, respectively. (b-c) Expanded view of the free energy profiles indicating the folding free energy barrier for GB1p in water and MeOH/water, respectively (the expanded view for GB1p in EMIM-Cl/water is not presented because of the unsuccessfully folding in the corresponding solution).

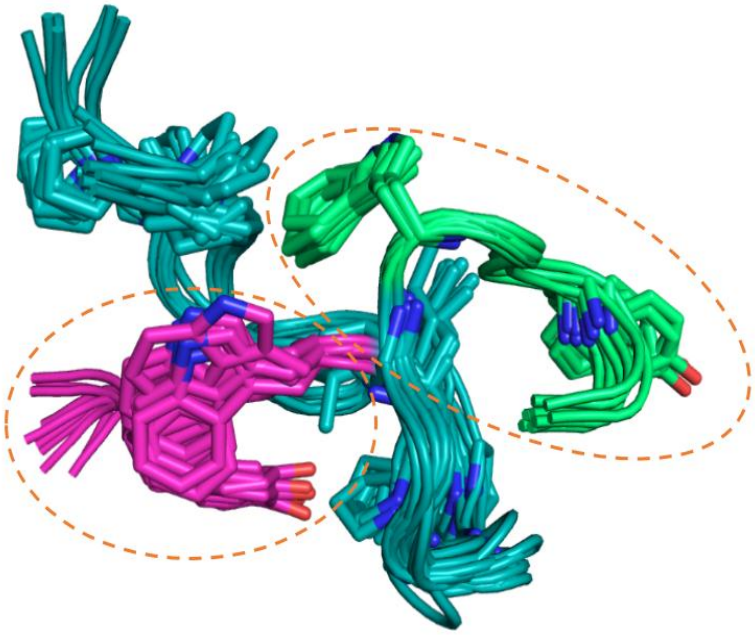

Figure S10. Comparison of the representative conformations of the unfolded states of Trp-cage in MeOH/water solution (green) and water (pink). Major difference at N-terminus is highlighted with yellow circles.
